# Optimization of single strand DNA incorporation reaction by Moloney murine leukaemia virus reverse transcriptase

**DOI:** 10.1093/dnares/dsy018

**Published:** 2018-06-11

**Authors:** Yoshiyuki Ohtsubo, Haruna Sasaki, Yuji Nagata, Masataka Tsuda

**Affiliations:** Department of Molecular and Chemical Life Sciences, Graduate School of Life Sciences, Tohoku University, Sendai, Japan

**Keywords:** adaptor ligation, template-independent DNA addition, DNA polymerase, reverse transcriptase

## Abstract

In this study, we investigated CIS reaction (clamping-mediated incorporation of single-stranded DNA with concomitant DNA syntheses) of Moloney murine leukaemia virus reverse transcriptase (MMLV-RT), and established a set of conditions with which single-stranded DNA is ligated to a G-tailed model substrate DNA at efficiencies close to 100%. Prior to the CIS reaction, a target blunt-end DNA was 3′ G-tailed by MMLV-RT in the presence of a tailing enhancer, deoxycytidine. In the CIS reaction, the G-tail reacted with a single-stranded DNA carrying a stretch of Cs on its 3′ end (termed as GAO for guide adaptor oligonucleotide), and MMLV-RT performed DNA polymerization, starting from the 3′ overhang, using the GAO as a template. We could append a given nucleotide sequence of as long as 72 nucleotides, which would be sufficient for various NGS-sequencing platforms. The high efficiency and the unique features of this MMLV-RT activity that enables the labelling of each DNA molecule with a unique degenerate sequence as a molecular identifier has many potential uses in biotechnology.

## 1. Introduction

Various fundamental DNA-handling techniques using the enzymatic activities of restriction enzymes, ligases, kinases, phosphatases, and DNA polymerases have been developed and combined for different biotechnological purposes.^1^ The addition of an enzymatic activity that catalyses a novel reaction would increase the applicability of the currently available genetic tools and establish a base on which a number of new methodologies could be founded.

Reverse transcriptases (RT) from different origins such as human immunodeficiency virus Type-1 and Moloney murine leukaemia virus (MMLV),^2^ has been shown to have clamping activity, which stabilizes a short duplex of dinucleotide at the 3′ tail of a double-stranded DNA and the 3′ end of a single-stranded DNA.^3–5^ The clamping activity enables RT-mediated DNA synthesis of the complementary strand of the single-stranded DNA, as well as the strand displacement synthesis originating from the single-stranded DNA.^3^ However, the efficiency of the overall reaction (hereafter CIS reaction; clamping-mediated incorporation of single-stranded DNA with concomitant DNA syntheses) have been low, leaving a high fraction of DNA unreacted,^3^ and thereby leaving its potential as a biotechnological tool unrealized.

MMLV-RT has been shown to catalyse a range of unique reactions; one such being the ‘template switching’ reaction that has been utilized for the efficient cloning of cDNA.^6^^,^^7^ The template switching occurs when the reverse transcribing MMLV-RT reaches the 5′ end of the RNA where it preferentially adds, in a template-independent fashion, a few dCMPs to the 3′ end of the newly synthesized cDNA. Then, in the presence of a single-stranded DNA (ssDNA) with a stretch of guanosine monophosphates (GMPs), referred to as a template switching oligo (TSO), MMLV-RT switches the template from RNA to the TSO and continues to incorporate deoxyribonucleotides that are complementary to the TSO, thereby extending the cDNA strand. Upon reaching the TSO end, MMLV-RT appends another 3′ tail consisting of dCMPs, to which another TSO (carrying 3′ GMPs) is incorporated, resulting in a concatenated end.^8^

MMLV-RT also has unusually strong tailing activity. In a previous study, we had optimized the tailing reaction conditions and shown that MMLV-RT appends to blunt double-stranded DNA ends with a 3′ tail of A, C, G, or T residues in a template-independent fashion.^9^ We also identified specific compounds that enhance C-, G-, and T-tailing reactions, thus enabling the appending of a tail composed of ∼3 Cs and Ts and 4–5 Gs.^10^ For the A-tailing reaction, specific enhancers have not been found but MMLV-RT inherently appends up to ∼4 As. Importantly, most of the DNA molecules in the reaction mixture are tailed, apparently leaving no DNA substrates unreacted.

It has not been addressed whether the template switching reaction of MMLV-RT involves a dynamic process, in which MMLV-RT molecule that has just finished reverse transcription (or DNA-dependent DNA polymerization) with its specific protein dynamics exerts the tailing and concomitant switching reaction. In addition, it has not been clear whether the performing of the tailing reaction by an MMLV-RT molecule is prerequisite for it to conduct subsequent template switching. The low efficiency of the reported CIS-reaction,^3^ might be due to the lack of those dynamic processes, or to lack of stable interaction between the tail and incoming single-stranded DNA molecule (hereafter GAO; guide adaptor oligonucleotide), or to the intrinsic nature of MMLV-RT that can efficiently conduct CIS reaction on RNA-DNA hybrid end but not on double-stranded DNA end.

As we could extend the tail lengths,^9^^,^^10^ we tested, in this study, the possibility that the extended tail increase the CIS reaction efficiency. Since this was the case, to establish a fundamental DNA manipulation technique to append a DNA of given nucleotide sequence to a blunt-end DNA, we sought for the ideal CIS reaction conditions including the best combination of the 3′ tail and GAO nucleotides, and investigated whether longer tails result in higher efficiency, and investigated the length of GAO that can be efficiently incorporated. The efficiency of the CIS reaction using a model DNA substrate reached almost 100% suggesting it to be useful for different biotechnological applications.

## 2. Materials and methods

### 2.1. Tailing reactions

The tailing reactions were conducted based on our previous reports.^9^^,^^10^ Wild-type MMLV-RT (200 U/µl) was purchased from Nippon Gene (Japan). The reaction mixture contained, in a total volume of 10 µl, 100 fmols to 1 pmol substrate DNA; 50 mM Tris–HCl, pH 8.3; 75 mM KCl; 6 mM MgCl_2_; 2 mM DTT; 4 mM dATP, dCTP, dGTP, or dTTP; 4 mM MnCl_2_; and 50 U MMLV-RT. Reactions were carried out in PCR tubes using a thermal cycler (C1000; Bio-Rad, USA) at 30°C. To enhance tailing, 200 mM deoxycytidine (for G-tailing), 40% saturated concentrations of deoxyguanosine (∼5 mM; for C-tailing), or deoxyadenosine (∼20 mM; for T-tailing) was added. We always added MnCl_2_ just before adding the MMLV-RT and used fresh DTT. The tailing reaction was conducted at 30°C for 5–90 min and terminated by phenol/chloroform/isoamylalcohol extraction or by adding a salt solution for the subsequent purification of DNA using a DNA-binding column.

### 2.2. CIS reaction

The CIS reaction mixture contained, in a total volume of 10 µl, 100 fmol tailed DNA substrate, 0.25 mM each of dNTPs, 2 pmol GAO, 50 units of MMLV-RT, 50 mM Tris–HCl pH 8.3, 75 mM KCl, 6 mM MgCl_2_, and 2 mM DTT. The solutions for the DNA substrate and GAO were mixed and kept at room temperature (25°C) and added to the rest of the reaction mixture kept at the same temperature. After it was mixed by tapping the tube, the reaction mixture was incubated at 37°C.

### 2.3. Reagents

Streptavidin was purchased from Nacalai Tesuque (Japan) and dissolved in PBS buffer. T4 DNA polymerase was purchased from Takara (Japan).

### 2.4. Preparation of FAM70 and 33G0 to 33G4

FAM70 DNA was prepared as described previously.^9^ Briefly, a 5′-FAM-labelled primer was used to amplify a 300-bp fragment, and the fragment was digested by PvuII to generate a 70-bp blunt-end fragment bearing FAM at one 5′ end; the fragment was then purified by 15% polyacrylamide gel electrophoresis. The opposite 3′ end of the FAM-labelled strand was the tailing target, and the 5′ end adjacent to the tailing target carried a phosphate group.

Five types of oligonucleotides (SA606 and SA677-SA680, see Fig. 3) bearing FAM at the 5′ end were annealed with SA659, purified as described earlier, and designated as 33G0 to 33G4. The concentrations of FAM70 and 33G0 to 33G4 were determined by measuring FAM fluorescence with an Infinite 200 Fluorescence Spectrophotometer (Tecan, Switzerland) using a FAM-labelled oligonucleotide (SA560) as a standard.

### 2.5. DNA length analysis using a capillary sequencer and data analysis

The length analysis was performed as described previously.^9^^,^^10^ To 1 ml of HiDi formamide, 10 µl of GeneScan-500 LIZ Size Standard (Thermo Fisher Scientific, USA), which contains 16 fragments of known sizes, was added to prepare HiDi-LIZ500. To 12.5 µl of HiDi-LIZ500, 0.5 µl of a reaction mixture was added, or when purified DNA samples were analysed, DNA samples were diluted with water, and DNA not exceeding 0.5 fmol was added. The samples were heat denatured at 96°C for 1 min and analysed with a 3130xl Genetic Analyzer (Thermo Fisher Scientific, USA) with a 50-cm capillary array and POP7 polymer. The data obtained were analysed using TraceViewer software,^9^ and two LIZ bands were chosen to calibrate the electropherogram. Peak areas were determined using TraceViewer software, and GAO incorporation rates were calculated by dividing sum of peak area of CIS reaction products by total peak area. When peaks corresponding to concatenated products were observed, those peaks were also counted as CIS reaction products.

### 2.6. Preparation of a size control

FAM70 DNA and SA682 (5′-GACGTGTGCTCTTCCGATCTCCCCCC**CTGTCTCTTATACACATCTGACGC**-3′; nucleotides complementary to the FAM70 end are in bold type, and intervening Cs are underlined) were mixed and annealed by heating and cooling and then extended by ExTaq DNA polymerase (TAKARA, Japan) for 10 min at 50°C. The product was used as a template for PCR amplification using the primers SA560 (FAM-AATGATACGGCGACCACCGAGATCTACAC-3′) and SA574 (5′-GACGTGTGCTCTTCCGATCTCCCCCC-3′) to amplify a 96-bp DNA fragment, which served as a size control.

## 3. Results

### 3.1. Schematic representation of CIS reaction

**Figure 1.**
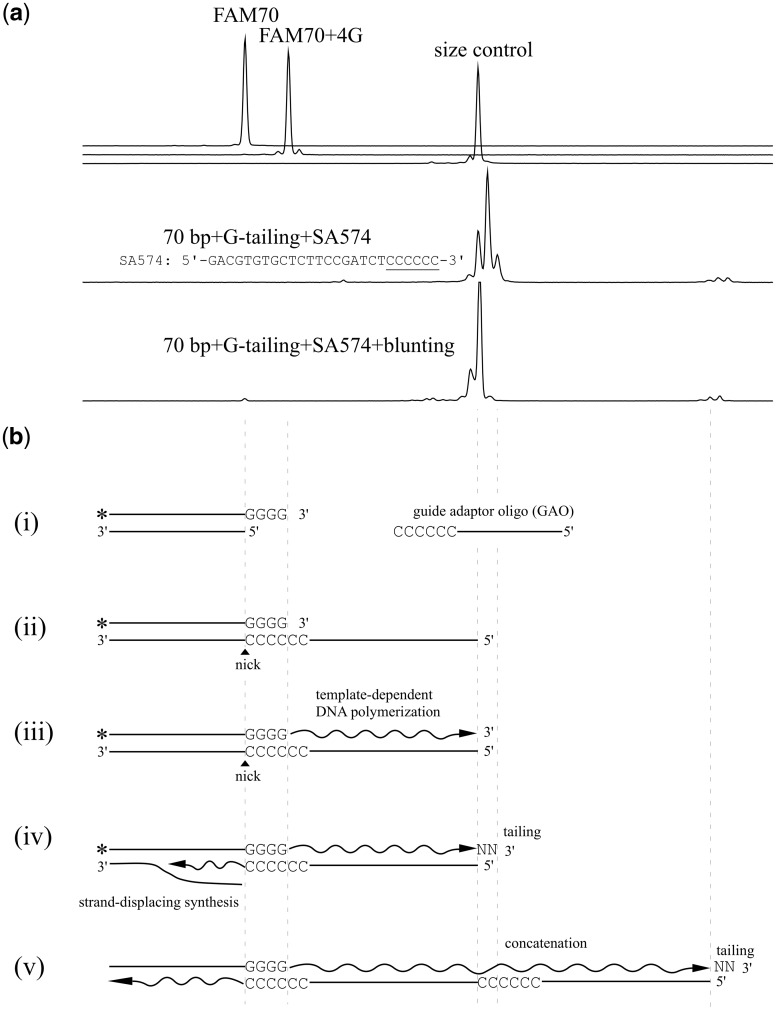
An example of CIS reaction and schematic interpretation. (a) Electropherograms obtained by analysing the samples by a capillary sequencer are shown; *x*-axis represents the retention time and *y*-axis represents the fluorescence signal intensity. FAM70 DNA was G-tailed, and reacted with GAO SA574, and the product was further subjected to blunting by KOD DNA polymerase. The size control was prepared by PCR (See materials and methods). (b) Schematic interpretation of the CIS reaction in panel (a). Asterisks indicate FAM label and ‘N’ indicate a nucleotide. (i) Before reaction, (ii) GAO annealing to the 3′ tail consisted of Gs, (iii) extension from the 3′ end of the G-tail by MMLV-RT, (iv) template-independent addition of nucleotides, and strand-displacing synthesis by the MMLV-RT, and (v) concatemer formation. The two panels share the same *x*-axis.

In this study, we explored the optimal CIS reaction conditions, and the highest efficiency close to 100% was observed when extensively G-tailed dsDNA was reacted with a GAO carrying a 3′ stretch of Cs, which is contrasted to the template switching reaction (see above). Figure 1a shows an example of the CIS reaction that was conducted with the optimized protocol, and Fig. 1b shows a schematic representation of the reaction, with the horizontal scale applicable to both panels. In this example, 70-bp blunt-end DNA, carrying the FAM fluorophore on one of its 5′ ends (FAM70), was subjected to G-tailing, resulting in a DNA product that mostly carried a tail containing four Gs (see the major peak denoted as ‘FAM70 + 4G’ with two minor accompanying peaks of +3G and +5G). In the CIS reaction, a GAO annealed with the 4G-tail, and MMLV-RT catalysed DNA polymerization to synthesize the complementary strand of the GAO. Upon reaching the GAO end, MMLV-RT appended a 3′ tail through its tailing activity, resulting in a 3′-overhang in a template-independent fashion (see the two peaks marked with ‘+1’ and ‘+2’ that are 1 and 2 nucleotides larger than the size control, respectively). To the newly appended tail, another GAO was incorporated, resulting in a concatenated end (marked with *). In the reaction, the 3′ end of the GAO was used for strand-displacing synthesis.

After blunting of the product, the peaks shifted to the left, and the size of the largest peak matched that of the size control, demonstrating that a tail 1–2 nucleotides long had formed (see discussion for the other accompanying peaks). Notably, most of the DNA ends were appended with the GAO, as indicated by a tiny peak at the position of FAM70 (see the products of the blunting reaction in panel A).

### 3.2. CIS reaction conditions

The CIS reaction was initiated by mixing two solutions at room temperature (25°C); one contained pre-tailed 100 fmol of DNA substrate and 2 pmol GAO and the other contained the rest of the reaction components, including MMLV-RT, dNTPs, and DTT. After mixing, the samples were transferred to 37°C and incubated. This protocol gave satisfactory results (see below) and was used as a standard protocol throughout this study.

### 3.3. G- and C-tailed DNA ends are good CIS reaction substrates

**Figure 2.**
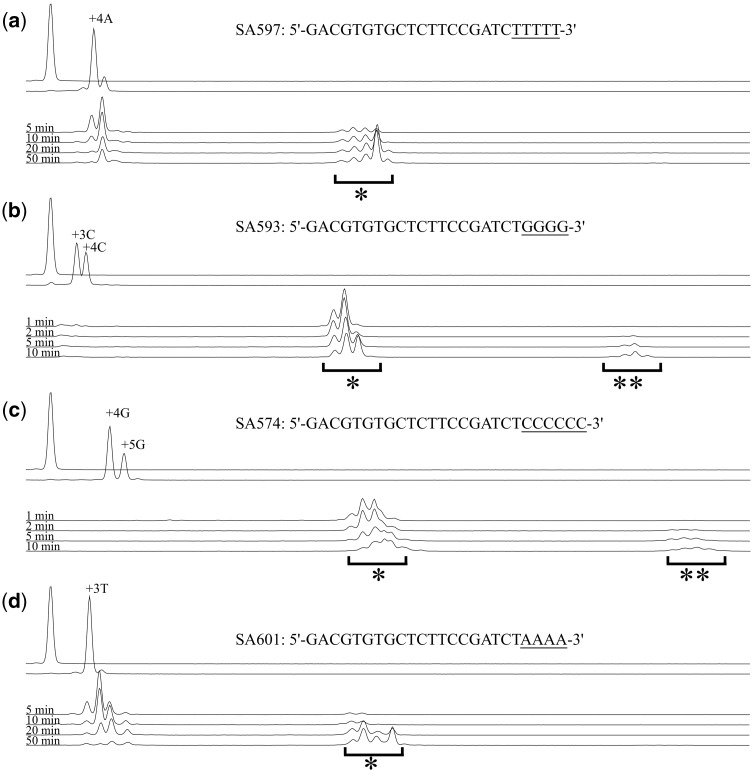
In the CIS reaction, G- or C-tailed DNA acquired the GAO efficiently. FAM70 DNA (a 70-bp dsDNA carrying FAM at one 5′ end) was subjected to A-, C-, G-, or T-tailing and then purified by phenol/chloroform/isoamylalcohol extraction and ethanol precipitation. The tailing reactions produced tails of different lengths; some of the peaks are labelled with the number of nucleotides added. Each of the tailed DNAs was reacted with the indicated GAO carrying a stretch of nucleotides complementary to the tail (underlined). At different time points, a portion of the reaction mixture was taken for analysis under denaturing conditions to investigate the length of the FAM-labelled strand. The panels present the results of A-tailing (a), C-tailing (b), G-tailing (c), and T-tailing (d) of FAM70 DNA and the subsequent CIS reaction with SA597 (a), SA593 (b), SA574 (c), and SA601 (d). In each panel, the electropherogram of unreacted FAM70 DNA and the tailing reaction product are also shown. Single asterisks (*) indicate the CIS reaction products, and double asterisks (**) indicate concatenated products. The CIS reaction products were observed as multiple peaks, which resulted from additional tailing reactions. Note that the sampling time points are different in panels (a, d) and (b, c).

To find the best combination of nucleotides for dsDNA 3′ tail and GAO 3′ end, we evaluated the efficiency with which A-, C-, G-, or T-tailed dsDNA was appended with a GAO carrying a stretch of Ts, Gs, Cs, or As at its 3′ end, respectively. In the first step of the experiment, a 70-bp blunt-end DNA carrying the FAM fluorophore at one of its 5′ ends (FAM70; the first line in each panel of Fig. 2) was subjected to A-, C-, G-, or T-tailing (see the second line in each panel of Fig. 2 for tail length distributions). Each tailing reaction was terminated by phenol/chloroform/isoamylalcohol extraction and ethanol precipitation. In the second step, the tailed-DNA was reacted with the GAO carrying a stretch of nucleotides complementary to the tail. The lengths of the complementary nucleotides were 4 for A, T, and G and 6 for C. The longer C-stretch was used to respond to the longer G-tail that exceeded four nucleotides long. Each reaction product was analysed with a capillary sequencer, and the resulting electropherogram was analysed using TraceViewer software.

As shown in Fig. 2, all types of tails analysed reacted with the corresponding GAO; however, regarding the reaction speed and yield, C- and G-tailed dsDNAs reacted well with the GAO, and A- and T-tailed dsDNAs reacted far less efficiently. For example, in our experimental setup using 100 fmol G-tailed DNA and 50 units of MMLV-RT in a 10-µl reaction, the GAO was appended to nearly 100% of G-tailed dsDNAs within a minute. In contrast, 30% of A-tailed DNAs remained unreacted even after 50 min of incubation (sum of multiple peak areas corresponding to the CIS products was divided by the total peak area). Moreover, G-tailed DNA reacted slightly better than C-tailed DNA [see weak signals representing unreacted substrates in panel (b)], and yielded less concatenated products [marked with double asterisks (**) in Fig. 2].

### 3.4. A longer 3′-tail is favourable for the CIS reaction

**Figure 3.**
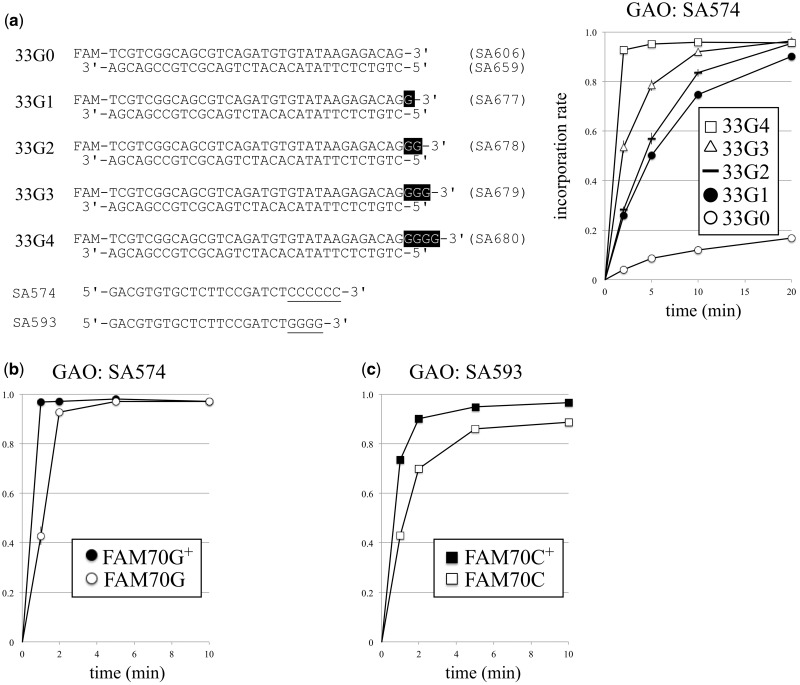
Longer tails are favourable for the CIS reaction. (a) Double-stranded DNAs 33G0 to 33G4, which are 33 base pairs long plus a 3′ tail of 0–4 Gs (carries FAM at the 5′ end of the G-tail strand), were subjected to the CIS reaction with GAO SA574. At the different time points, a portion of the reaction mixture was taken for analysis using a capillary sequencer, and the GAO incorporation rate was calculated as described in the materials and methods. (b) FAM70 DNA was G-tailed in the presence (FAM70G^+^) or absence (FAM70G) of the tailing enhancer deoxycytidine, purified by phenol/chloroform/isoamylalcohol extraction and ethanol precipitation, and then reacted with SA574. (c) FAM70 DNA was C-tailed in the presence (FAM70C^+^) or absence (FAM70C) of the tailing enhancer deoxyguanosine, purified, and reacted with GAO SA593. Values are the average of triplicate experiments, and standard deviations are shown.

To determine whether a longer 3′ tail is favourable for the CIS reaction, 33-bp dsDNAs with 3′ G-tails 0–4 nucleotides long (33G0 to 33G4; see Fig. 3a) were reacted with GAO SA574. The results shown in Fig. 3a clearly indicate that the longer the tail was, the higher the efficiency. To further clarify this, FAM70 DNA was G- or C-tailed in the presence (marked FAM70G+ and FAM70C+ in Fig. 3b and c, respectively) or absence of a cognate tailing-enhancer,^10^ and the tailed product was reacted with a corresponding GAO (Fig. 3b and c). Again, longer 3′ tails were more favourable for CIS reaction.

### 3.5. Ribonucleotides at the GAO 3′ end facilitate the CIS reaction

In template switching reactions, oligonucleotides with a few ribonucleotides^11^ or locked-nucleotides^12^ at their 3′ end are often used to improve the reaction efficiency, possibly by increasing the thermostability between the tail and the 3′ end of the oligonucleotides. To assess the effect of replacing the 3′ deoxyribonucleotides of GAO with ribonucleotides on the CIS reaction, FAM70 DNA tailed with C or G in the absence of enhancers was reacted with a GAO that carried rGrGrG or rCrCrC at its 3′ end (the lower case ‘r’ indicates ‘ribonucleotide’).

**Figure 4.**
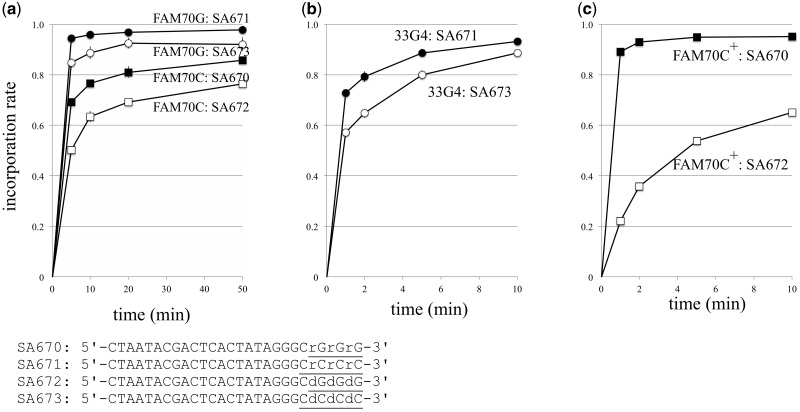
GAOs carrying 3′ ribonucleotides react more efficiently. G-tailed FAM70 DNA (G2^98%^) or C-tailed FAM70 DNA (C1^82%^C2^12%^C3^5%^) prepared by a tailing reaction conducted in the absence of a tailing enhancer (a), 33G4 DNA (b), and C-tailed FAM70 DNA (C3^56%^C4^44%^) in the presence of the tailing enhancer deoxyguanosine (c) were reacted with the indicated GAOs. Values are the average of triplicate experiments, and standard deviations are shown. SA670 and SA672 are equivalent to TS-oligo-rG3 and TS-oligo-dG3, respectively, in reference.^6^

Figure 4a shows that GAO with ribonucleotides reacted more efficiently than its deoxynucleotidyl counterpart, i.e. G-tailed DNA (FAM70G) reacted faster with SA671 (carrying dCrCrCrC at its 3′ end) than with SA673 (carrying dCdCdCdC at its 3′ end), and C-tailed DNA (FAM70C) reacted faster with SA670 (carrying rGrGrG at its 3′ end) than with SA672 (carrying dGdGdG at its 3′ end). The superiority of the ribonucleotidyl GAO to deoxyribonucleotidyl GAO was also observed when a dsDNA with a longer G-tail was tested (Fig. 4b). A tailed 33G4 DNA (carrying four Gs as a tail) reacted faster with SA671 than with SA673. Likewise, FAM70 DNA subjected to C-tailing in the presence of an enhancer (C3, 55%; C4, 44%) reacted faster with SA670 than with SA672 (Fig. 4c).

### 3.6. 5′ biotin on GAO inhibits concatenation

Because GAO concatenation might compromize the downstream application of CIS reaction products, its suppression is desirable. GAO concatenation might also arise through the annealing of two GAOs and complementary strand synthesis by MMLV-RT and subsequent tailing and the CIS reaction.

**Figure 5.**
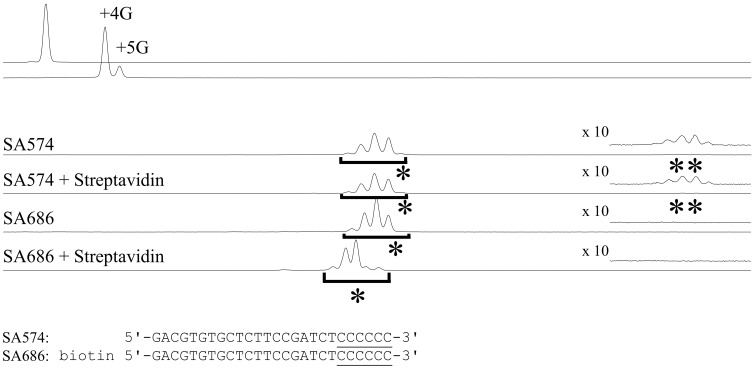
Biotin labelling at the GAO 5′ end prevented concatenation. FAM70 DNA was G-tailed (G4^80%^G5^20%^) and purified using a DNA purification column. Two types of GAOs, SA574 and SA686, with SA686 bearing a biotin moiety at its 5′ end, were reacted with the G-tailed DNA for 20 min in the presence or absence of 10 pmol streptavidin. The single asterisk (*) indicates the CIS reaction product, and parts of the data were magnified 10-fold to show the concatenated products (**). Note that, under the denaturing conditions of the capillary sequencer, the FAM-labelled strand is dissociated from the biotinylated strand.

We found that 5′-biotinylated GAO (SA686) suppressed the concatenation (Fig. 5). In Fig. 5, when unbiotinylated GAO (SA574) was used, peaks representing the concatenated products were observed (marked with ** in Fig. 5). In contrast, when biotinylated GAO (SA686) was used, no concatenated products were observed. Originally, we expected that the addition of streptavidin to the reaction mixture containing biotinylated GAO would lead to the formation of a macromolecular complex that might exclude the MMLV-RT from the 5′ end of the GAO by steric hindrance, thereby inhibiting the concatenation. Contrary to our expectation, even in the absence of streptavidin, biotinylated GAO suppressed the concatenation. However, the addition of streptavidin to the reaction using SA686 changed the peak profile of the CIS reaction product (Fig. 5), indicating that the strand synthesis of the last few bases was suppressed by the biotin-streptavidin complex.

### 3.7. Fate of the GAO in the CIS reaction

**Figure 6.**
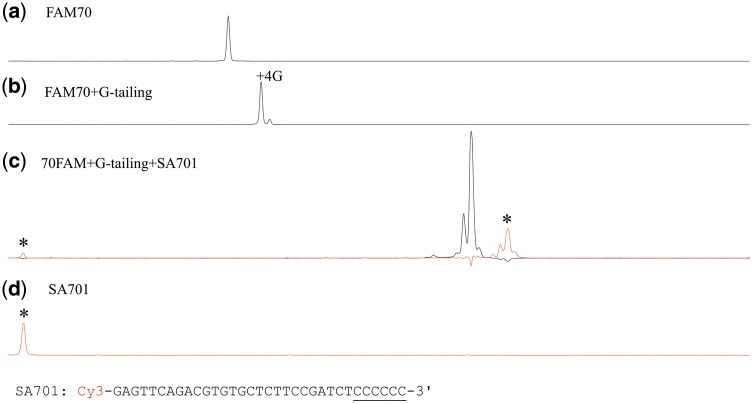
GAO is incorporated into the target DNA end. FAM70 DNA (a) was G-tailed (G4^89%^G5^11%^) in the presence of the enhancer deoxycytidine (b) and then reacted in a CIS reaction with GAO SA701, which carries a Cy3 label at its 5′ end. The CIS reaction product was purified by polyacrylamide gel electrophoresis and then analysed (c). As a size control, SA701 was also analysed (d). The Cy3 signals are indicated by asterisks.

In this study, we confirmed the strand displacement synthesis by RTs that originates from the 3′ end of GAO followed by the clamping of the 3′ tail of dsDNA and 3′-end nucleotides of the single-stranded DNA.^3^ A GAO (SA701) with a Cy3-labelled 5′-end was reacted with a G-tailed DNA and then analysed to see the extended Cy3-labelled fragments. To exclude the unreacted GAO, the reaction product was subjected to polyacrylamide-gel electrophoresis, and the band with the expected size of the CIS reaction product was excised, purified, and analysed with a capillary sequencer under denaturing conditions. As shown in Fig. 6, the GAO strand was extended as demonstrated by the appearance of peaks that were located close to the peaks for the CIS reaction product of the FAM-labelled upper strand. The slight difference in the electrophoretic mobilities of FAM and the Cy3-labelled strand might reflect the different nucleotide compositions of the strands. The peak area ratio of the Cy3 to FAM signals was close to the ratio of 0.25 obtained when running equimolar Cy3 or FAM-labelled single-stranded DNA (data not shown), indicating that most of the GAO molecules were incorporated into the target DNA that underwent strand extension. Because MMLV-RT has not been reported to have 5′ -> 3′ exonuclease activity, this extension might represent the strand-displacing synthesis that MMLV-RT catalyses during cDNA synthesis.

The extension from the 3′ end of the GAO is not necessary for the CIS reaction because a GAO with a 2′, 3′-dideoxy end was incorporated at a rate very similar to that of a GAO with a 2′ deoxy end (data not shown).

### 3.8. GAO lengths and efficiencies

**Figure 7.**
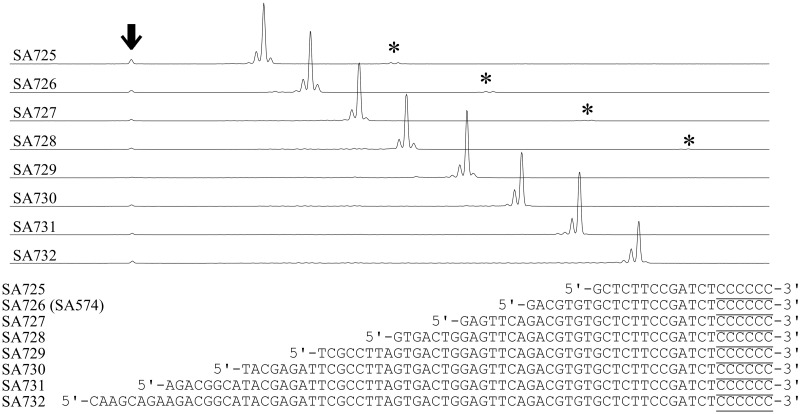
G-tailed DNA end efficiently acquired GAOs of different lengths. G-tailed FAM70 DNA (G4^80%^G5^20%^) was reacted with GAOs SA725 to SA732 of different lengths for 5 min and then purified using DNA purification columns. The purification product was further subjected to blunting by T4 DNA polymerase and then analysed. Asterisks indicate the concatenated products, and the arrow indicates the peaks of the original size (70 nucleotides).

In practical use, GAOs with different lengths might be to be incorporated. To investigate the effect of GAO length on CIS reaction efficiency, eight types of GAOs with different lengths ranging from 19 to 72 nucleotides long were reacted with G-tailed FAM70 DNA (G4^80%^G5^20%^). As shown in Fig. 7, GAO length did not seem to affect the reaction efficiency, and 98% of the DNA ends underwent the CIS reaction.

### 3.9. Concentrations of G-tailed substrate and efficiencies

**Figure 8.**
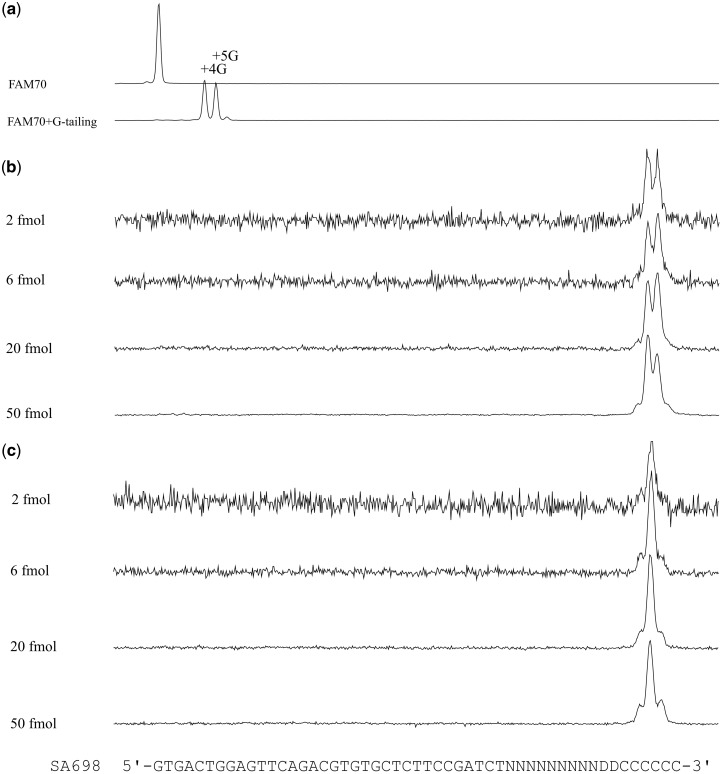
CIS-reactions conducted under different substrate DNA concentrations. (a) FAM70 DNA and FAM70 DNA subjected for G-tailing (G4^50%^G5^46%^G6^4%^) used as a substrate in panels (b) and (c). In total, 2–50 fmol (0.2–5 nM) of G-tailed FAM70 DNA were reacted with 2 pmol (b) or 0.5 pmol (c) of GAO SA698, which carries N9D2 representing a molecular identifier (N: A or C or G or T; D: A or G or T) for 2 min and then analysed. In panels (b) and (c), electropherograms were *y*-axis scaled to enable to see the absence of signals from unreacted CIS reaction substrate. Three panels share the same *x*-axis.

A series of concentrations of a G-tailed DNA ranging from 0.2 nM (2 fmol in 10 µl reaction) to 5 nM (50 fmol in 10 µl reaction) was reacted with 0.5 or 2 pmol of GAO, for 2 min, and the reaction products were analysed. For all the samples, we observed only CIS reaction product peaks, demonstrating that the CIS reaction could be carried out efficiently under a wide range of DNA concentrations (Fig. 8).

## 4. Discussion

In this study, upon our finding of tailing enhancers,^10^ we optimized the CIS reaction, which has been reported but could not be conducted at a high efficiency. By applying the best set of conditions, adaptor DNA sequence could be appended to the double-stranded blunt DNA end at efficiency close to 100% that no other DNA-handling techniques have achieved.

### 4.1. Accompanying peaks

When the CIS reaction products were subjected to treatment with T4 DNA polymerase, one major peak was associated with the peaks of products that were a few nucleotides shorter or one nucleotide longer (Fig. 7). The shorter peaks might represent insufficient extension by T4 DNA polymerase. To support this idea, although at a lower level, 3′ recessed ends were observed for the product of the PCR reaction using KOD DNA polymerase, which is supposed to generate blunt ends (see the size control in Fig. 1a). The other possibility is the ‘erosion’ of the GAO 3′ end during storage (we stored GAOs at 4°C in TE buffer). To assess this possibility, after long-term storage at 4°C for 6 months, some, although significant, fractions of the GAO were found to have undergone erosion of a few nucleotides, which was determined by measuring the mass spectra of the stored oligonucleotides (data not shown). Another possibility is that a proportion of the GAO was not synthesized as its full length. In contrast, the longer product might be the result of the gapped-annealing of GAO that increased the size of the product by the length of the gap. Although the gaps between GAO and dsDNA have been shown to decrease the reaction efficiency considerably,^4^ extended G-tail of c.a. four nucleotides long might stabilize the gapped annealing.

### 4.2. Importance of tailing enhancers

In our previous study, we identified enhancers for C-, G-, and T-tailing reactions.^10^ The results of this study clearly showed that the length of the tail is important for the efficiency of the CIS reaction (Fig. 3). Because MMLV-RT can append up to four dAMPs as a tail even in the absence of an enhancer,^9^ when the G-tail length of the target DNA is short and CIS reaction was started in the presence of dNTPs, MMLV-RT can append the G-tail with a few additional deoxyadenosine monophosphates, resulting in a mosaic tail that is not complementary, and hence not reactive to the GAO that carry Cs at it 3′ end. In this regard, the addition of four or more Gs is desirable because no additional A would be further appended to such a long G-tail. In addition, the removal of enhancers as well as Mn^2+^ ions, which are also enhancers,^8^^,^^9^^,^^13^ after the tailing reaction is important for a successful CIS reaction.

The removal of enhancers also seems to be important for preventing concatemer formation. Concatemer formation was noted in the experiment shown in Fig. 2c but not in that shown in Fig. 5, and this difference might have resulted from the different methods used to purify DNA after the tailing reaction (phenol/chloroform/isoamylalcohol extraction and ethanol precipitation in Fig. 2 and a DNA purification column in Fig. 5).

### 4.3. Use of a GAO with 3′ ribonucleotides

In this study, we showed that even with a long tail of Gs up to four nucleotides long, a GAO carrying 3′ ribonucleotides reacted more efficiently than a GAO carrying the deoxyribonucleotidyl counterpart. However, in practical use, the advantage of a ribonucleotidyl GAO seems to be limited, especially when extensively G-tailed DNA is subjected to the CIS reaction. In Fig. 4a, substrates G-tailed in the absence of enhancer (mostly +G2) was used, and the rG-carrying GAO exhibited only ∼5% higher efficiency when compared with GAO that carry dG. In addition, 98% of reaction yield can be achieved even when deoxyribonucleotidyl GAOs are used when extensively G-taild DNA (mostly +G4) was a substrate (Fig. 7). The chemical instability as well as the increased cost for ribonucleotidyl GAO preparation also supports the use of deoxyribonucleotidyl GAO.

From a mechanistic point of view, the 3′ ribonucleotides of the GAO appear to not only increase the thermostability with the deoxyribonucleotidyl tail but also stably interact with MMLV-RT, thereby increasing the efficiency. This idea is consistent with the fact that the physiological substrate of MMLV-RT is not DNA but RNA.

### 4.4. Effect of the GAO sequence on CIS reaction efficiency

We used two types of GAOs: one represents the adaptor sequence of the Illumina sequencer (used here except in Fig. 4)^6^ and the other represents single-stranded DNAs as used by Chenchik et al.^6^ (used in Fig. 4; SA670-SA673). Not only the 3′ end of the GAO but also the entire nucleotide sequence affect the CIS reaction efficiency (for example, compare the SA673 data in Fig. 4b and the SA574 data in Fig. 3a). The structure that each GAO can have, e.g. dimers formed between two GAO molecules at the reaction temperature, may affect the efficiency. We also observed a low CIS reaction efficiency with a GAO with six Gs at its 3′ end, although the reason for this was unclear (data not shown). Keeping these observations in mind, each GAO to be incorporated should be examined prior to practical use in a system like the one we used in this study.

### 4.5. Use of a biotin-labelled GAO and streptavidin to prevent concatenation

Concatemer formation on the cDNA ends of template-switching oligonucleotides has been demonstrated and proposed as a reason for the high background and low cDNA yield, especially when a small amount of RNA is used for cDNA synthesis.^8^ In their study, Kapteyn et al. demonstrated the usefulness of nucleotide isomers that form non-standard base pairs to reduce concatemer formation. Our findings indicated that more common 5′ end modifications on the GAO, e.g. biotinylation, prevented concatenation and that combining with streptavidin further prevented additional tailing (Fig. 5), suggesting that steric hindrance is sufficient to prevent concatenation.

### 4.6. CIS reaction efficiency

When we analysed the CIS reaction products, we often observed no peaks representing the initial DNA targets (see Fig. 1a for an example). However, after T4 DNA polymerase treatment, a small but distinct peak with the size of the untailed DNA substrate was always observed. This peak indicated that some fractions of MMLV-RT failed to complete the synthesis of the strand complementary to the GAO, leaving an extended tail resulting from the incomplete synthesis that was removed by T4 DNA polymerase. This failure might have been because the dGTP solution used for tailing was not 100% pure, resulting in a base-mismatch between the tail and 3′ end of the GAO. In support of this conjecture, RT’s high error rate has been suggested to be due to its efficiency at extending mismatches.^14^^,^^15^

Nonetheless, CIS reaction efficiency is very high and might replace the traditional single-A-tailing followed by adaptor DNA ligation by DNA ligase, for which efficiencies were analysed by droplet digital PCR assays and reported to be 3–20%.^16^

## 5. Conclusions

Adaptor ligation to DNA ends is used as a fundamental DNA technique in library preparations for PacBio,^17^ Illumina,^18^ and NanoPore^19^ sequencing technologies for various research objectives such as *de novo* genome sequencing, re-sequencing for mutation mapping, and transposon mutant library analysis.^20^ Our method termed the CIS reaction is advantageous over existing methods in that the CIS reaction can label each DNA at a high efficiency, thereby facilitating analyses for which only limited amount of DNA is available. In addition, when using the CIS reaction with a GAO carrying a random sequence, it is easy to label each DNA fragment with a unique molecular identifier^21–23^ before the PCR-amplification step, thereby enabling the quantitative processing of NGS reads.
